# Deceleration capacity of heart rate predicts 1‐year mortality in patients undergoing transcatheter edge‐to‐edge mitral valve repair

**DOI:** 10.1002/clc.24007

**Published:** 2023-03-22

**Authors:** Lars Mizera, Dominik Rath, Jürgen Schreieck, Peter Seizer, Meinrad Paul Gawaz, Martin Duckheim, Christian Eick, Karin A. L. Müller

**Affiliations:** ^1^ Department of Cardiology and Angiology University of Tübingen Tübingen Germany; ^2^ Department of Cardiology and Angiology Ostalbklinikum Aalen Aalen Germany

**Keywords:** cardiac autonomic dysfunction, deceleration capacity, MitraClip, mortality

## Abstract

**Background:**

Risk stratification for transcatheter procedures in patients with severe mitral regurgitation is challenging. Deceleration capacity (DC) has already proven to be a reliable risk predictor in patients undergoing transcatheter aortic valve implantation. We hypothesized, that DC provides prognostic value in patients undergoing transcatheter edge‐to‐edge mitral valve repair (TEER).

**Methods:**

We retrospectively analyzed electrocardiogram signals from 106 patients undergoing TEER at the University Hospital of Tübingen. All patients received continuous heart‐rate monitoring to assess DC following the procedure. One‐year all‐cause mortality was defined as the primary end point.

**Results:**

Sixteen patients (15.1%) died within 1 year. The DC in nonsurvivors was significantly reduced compared to survivors (5.1 ± 3.0 vs. 3.0 ± 1.6 ms, *p* = 0.002). A higher EuroSCORE II and impaired left ventricular function were furthermore associated with poor outcome. In Cox regression analyses, a DC < 4.5 ms was found a strong predictor of 1‐year mortality (hazard ratio: 0.10, 95% confidence interval: 0.13–0.79, *p* = 0.029). Finally, a significant negative correlation was found between DC and residual mitral regurgitation after TEER (*r* = −0.41, *p* < 0.001).

**Conclusion:**

In patients with severe mitral regurgitation undergoing TEER, DC may serve as a new predictor of follow‐up mortality.

## Graphical abstract

In patients with severe mitral regurgitation undergoing transcatheter edge‐to‐edge mitral valve repair, deceleration capacity (DC) may serve as a new predictor of follow‐up mortality.

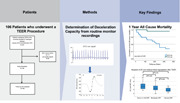

**Abbreviations**
ANP: atrial natriuretic peptide
DC: deceleration capacity
ECG: electrocardiogram
EuroSCORE: European system for cardiac operative risk evaluation
FMR: functional mitral regurgitation
hs‐cTnI: high‐sensitivity cardiac troponin‐I
LA: left atrium
LV: left ventricle
LVEF: left ventricular ejection fraction
MR: mitral regurgitation
NYHA: New York Heart Association
PAPsys: systolic pulmonary arterial pressure
PRSA: phase‐rectified signal averaging
TAVI: transcatheter aortic valve implantation
TEER: transcatheter edge‐to‐edge mitral valve repair
TOE: transesophageal echocardiography
TTE: transthoracic echocardiography

## 1 BACKGROUND

Mitral regurgitation (MR) is the most common valve condition in industrialized countries in people aged >65 years.^1^ According to the 2021 ESC/EACTS Guidelines for the management of valvular heart disease, transcatheter edge‐to‐edge mitral valve repair (TEER) using edge‐to‐edge devices represents an efficacious treatment option for patients with chronic heart failure, severe functional mitral regurgitation (FMR) and prohibitive perioperative risk.^2^, ^3^ Both beneficial effects on patients’ symptoms reflected in improved New York Heart Association (NYHA) functional class as well as a significant reduction in rehospitalizations could be attributed to TEER.^4^, ^5^, ^6^ Reichart et al. demonstrated a correlation of residual MR after TEER with long‐term outcome in patients with FMR.^7^ However, according to the COAPT trial and MITRA‐FR, the impact of TEER on mortality yielded opposite results, emphasizing the importance of selection of appropriate candidates for this procedure.^5^, ^8^ Thus, reliable and validated risk stratification tools for predicting mortality in patients undergoing TEER are urgently needed. Deceleration capacity (DC) has already been proven to be an independent predictor of 1‐year mortality in patients with severe aortic stenosis undergoing transcatheter aortic valve implantation (TAVI).^9^ This parameter provides indirect insight into the balance of the autonomic nervous system and can be determined noninvasively and objectively by heart rate variability analysis. Autonomic impairment and reduced DC are already established as markers of cardiovascular risk and outcome.^10^, ^11^, ^12^, ^13^

This study aimed to evaluate the prognostic value of DC on mortality in patients with severe MR undergoing TEER.

## 2 METHODS

### 2.1 Study design and participants

In this study, we retrospectively enrolled 106 consecutive symptomatic patients with severe MR who underwent TEER at the University Hospital of Tübingen, Germany, between May 2010 and December 2015. Transthoracic (TTE) and transesophageal (TEE) echocardiography were performed to evaluate mitral valve morphology and MR severity before and at the end of the procedure. The assessment of MR severity followed the current European Association of Echocardiography guidelines.^14^ All patients were qualified for TEER by an interdisciplinary heart team. Written informed consent was obtained wherever possible.

History of atrial fibrillation (AF) was evaluated upon study inclusion. Patient, cardiac, and operation‐related factors of the logistic European System for Cardiac Operative Risk Evaluation (EuroSCORE) II were assessed.^15^ In addition, hemoglobin (Hgb) and creatinine (Cr) levels were recorded at baseline. Patients previously treated with TEER were not included. In addition, the presence of sinus rhythm was mandatory for inclusion in the study.

The study was approved by the institutional ethics committee (260/2015R).

### 2.2 Assessment of DC

We evaluated data from the surveillance monitors (DASH 4000/5000 and Teleguard [General Electric, Fairfield, CT]; sample frequency, 100 Hz) to determine the DC. Only patients with sinus rhythm were included in this study. The ECG recording should be of sufficient quality without noisy signals or various artifacts to allow automatic DC calculation from a 24‐h ECG Holter or monitor ECG. The technical details of automated assessment of DC from noisy and nonstationary ECG signals have been described in detail by Eick et al.^16^ Briefly, “anchor points” were defined on the RR interval series and processed by a mathematical algorithm called phase‐rectified signal averaging (PRSA). DC as central amplitude of the PRSA signal is subject to the influence of sympathetic and parasympathetic modulation.^17^ Sinus rhythm is mandatory in the calculation of DC. In previous studies, Bauer et al. postulated that an impaired DC ≤ 4.5 ms is associated with a higher risk of mortality.^10^

### 2.3 Outcome

All patients were followed‐up for 1 year after study enrollment. The primary endpoint was defined as all‐cause mortality 1 year after TEER. Follow‐up was conducted via our outpatient clinic and telephone interviews.

### 2.4 Statistical analysis

The entire statistical analyses were conducted using SPSS Version 26 (SPSS Inc.). Data were analyzed by paired Student *t*‐test and presented as mean ± standard deviation. Analyses included cross‐tabulation and Pearson's *χ*² tests, with *p*  <  0.05 defined as statistically significant. Associations between nonnormally distributed data were evaluated with Spearman's rank correlation coefficient (rho). Cox regression analyses were applied to analyze independent associations between all‐cause mortality and EuroSCORE II, left ventricular ejection fraction (LVEF), and DC. Furthermore, the prognostic significance of DC was analyzed by using Kaplan–Meier curves and log‐rank tests.

## 3 RESULTS

Between January 2010 and December 2015, 243 consecutive patients with high‐grade MR and higher risk for surgery underwent TEER at the University Hospital of Tübingen. Due to AF or low‐quality ECG signals, 106 patients could be considered for further analysis (supplementary file). One‐year follow‐up and survival analysis could be performed in all patients. Baseline characteristics of the study cohort are shown in Table 1. Sixteen patients (15.1%) died within the 1‐year observation period. Nonsurvivors presented with lower LVEF and increased EuroSCORE II at study inclusion. On the other hand, systolic pulmonary artery pressure (PAPsys) or Hgb levels were not found to be different when compared to survivors. Serum Cr concentration was significantly elevated, indicating a relevant kidney injury in nonsurvivors.

**Table 1:** Baseline characteristics of the overall cohort (*n* = 106).

Age, years (mean ± SD)	75 (±10)
Male, *n* (%)	64 (60.4)
Cardiovascular risk factors, *n* (%)	
Arterial hypertension	85 (80.2)
Diabetes mellitus	29 (27.4)
Atrial fibrillation, paroxysmal	47 (44.3)
Known coronary artery disease	80 (75.5)
Chronic kidney disease	57 (53.8)
Echocardiography	
Left ventricular function, %, mean (±SD)	39 (13)
RV pressure, mmHg, mean (±SD)	40 (±13)
Conventional risk variables	
EuroSCORE II, mean (±SD)	7.2 (5.9)
Laboratory values at admission, median (25th/75th percentile)	
Hemoglobin (mg/dL)	12.1 (10.8/13.1)
Leukocytes (1000/µL)	7.8 (6.2/9.0)
Creatinine (mg/dL)	1.2 (1.0/1.7)
GFR (mL/m²)	50 (39/67)
C‐reactive protein (mg/dL)	0.5 (0.1/1.3)
CK (U/L)	64 (46/102)
Medication at admission, *n* (%)	
Oral anticoagulation	42 (40.0)
ACEi/ARB	90 (84.9)
Diuretics	100 (94.3)
Calcium channel blockers	17 (16.0)
Beta blockers	89 (84.0)
Statins	64 (60.4)
ASS	54 (50.9)
P2Y12 inhibitors	36 (34.0)

**Figure 1:**
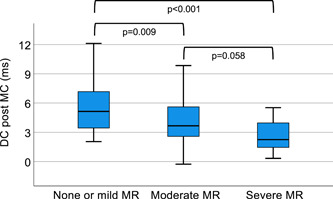
Boxplots of deceleration capacity and residual mitral regurgitation after transcatheter edge‐to‐edge mitral valve repair.

DC was found significantly reduced in patients with poor outcome (Figure 1). Nonsurvivors were also more likely to show impaired DC < 4.5 ms (Nonsurvivors 93.8% versus survivors 46.7%, *p* < 0.001). Considering the patients with DC > 4.5 ms, just one death occurred after 1 year. Table 2 compares baseline characteristics and outcome in nonsurvivors and survivors. A multivariable Cox regression analysis that included age, gender, use of beta blockers, DC, EuroSCORE II, LVEF, and serum level of Cr revealed that only DC was independently associated with all‐cause follow‐up mortality (Table 3).

**Table 2:** Baseline characteristics and deceleration capacity stratified according to mortality.

	Nonsurvivors	Survivors	
	(*n* = 16)	(*n* = 90)	*p*‐Value
Age, years (mean ± SD)	73 (±11)	75 (±10)	0.536
Male, *n* (%)	13 (81.3)	51 (56.7)	0.095
Cardiovascular risk factors, *n* (%)			
Arterial hypertension	12 (75.0)	73 (81.1)	0.517
Diabetes mellitus	7 (43.8)	22 (24.4)	0.135
Atrial fibrillation, paroxysmal	8 (50.0)	39 (43.3)	0.0786
Known coronary artery disease	12 (75.0)	68 (75.6)	1.000
Chronic kidney disease	11 (68.8)	46 (51.1)	0.277
Echocardiography			
Left ventricular function, %, mean (± SD)	30 (±10)	41 (±12)	**0.002**
RV pressure, mmHg, mean (± SD)	43 (±14)	39 (±13)	0.405
Conventional risk variables			
European system for cardiac operative risk evaluation II, mean (±SD)	10.4 (±6.8)	6.7 (±5.6)	**0.008**
Laboratory values at admission, median (25th/75th percentile)			
Hemoglobin (mg/dL)	11.7 (10.1/12.4)	12.2 (10.8/13.3)	0.086
Leukocytes (1000/µL)	7.9 (5.8/8.6)	7.7 (6.1/9.0)	0.640
Creatinine (mg/dL)	1.6 (1.3/2.1)	1.2 (1.0/1.6)	**0.015**
GFR (mL/m²)	42 (28/52)	52 (42/68)	0.060
C‐reactive protein (mg/dL)	0.9 (0.1/2.6)	0.5 (0.1/1.2)	0.217
CK (U/l)	55 (35/127)	65 (46/98)	0.723
Medication at admission, *n* (%)			
Oral anticoagulation	6 (37.5)	36 (40.0)	1.000
ACEi/ARB	12 (75.0)	78 (86.7)	0.398
Diuretics	15 (93.8)	85 (94.4)	1.000
Calcium channel blockers	2 (12.5)	15 (16.7)	1.000
Beta blockers	14 (87.5)	75 (83.3)	0.687
Statins	9 (56.3)	55 (61.1)	1.000
ASS	7 (43.8)	47 (52.2)	0.781
P2Y12 inhibitors	6 (37.5)	30 (33.3)	0.771
Main study parameter			
Deceleration capacity (ms)	3.0 (±1.6)	5.1 (±3.0)	**0.002**
Deceleration capacity ≤4.5 ms	15 (93.6)	42 (46.7)	**<0.001**

*Note*: Bold values indicate significance level of 0.05.

**Table 3:** Cox‐regression analysis with all‐cause mortality as dependent variable and age, gender, use of beta blockers, EUROSCORE II, left ventricular function, creatinine and deceleration capacity (DC) as independent variables.

	Regression coefficient (B)	Hazard ratio 95% confidence interval	*p*‐Value
Age	1.02	0.97–1.07	0.467
Gender	0.54	0.15–1.97	0.350
Antarrhythmic drugs (beta blockers)	3.26	0.36–29.24	0.291
EUROSCORE II	1.06	1.0–1.13	0.097
Left ventricular function	0.95	0.89–1.01	0.107
Creatinine	1.11	0.66–1.88	0.693
DC	0.70	0.51–0.96	**0.027**

*Note*: Bold values indicate significance level of 0.05.

**Figure 2:**
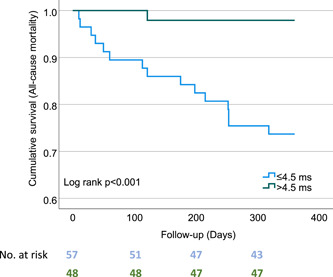
Kaplan–Meier curves for cumulative survival according to deceleration capacity.

In addition, our data revealed an inverse correlation between DC and residual MR (*r* = −0.41, *p* < 0.001) as shown in Figure 2.

## 4 DISCUSSION

The major findings of the current study of 106 patients with sinus rhythm and severe MR undergoing TEER may be summarized as follows: (1) Low DC was associated with 1‐year mortality. (2) In addition to well‐known predictive markers such as impaired LVEF or EuroSCORE II, DC was predictive of adverse outcomes and also reliably identified low‐risk patients. (3) Residual MR severity was inversely correlated with DC.

Among patients with chronic heart failure and FMR, the overall prognosis is poor and risk prediction in this multimorbid patient cohort with high surgical risk remains challenging.^18^ Our cohort represents the typical patient collective for interventional procedures with old age, impaired LVEF, and a high burden of comorbidities such as ischemic cardiomyopathy and pulmonary hypertension. Our findings confirm the predictive value of conventional risk predictors such as the ES II, LVEF, and renal failure 1 year after TEER.^19^, ^20^ Several studies found that TEER did not appear to alter disease progression regardless of the magnitude of reduction in MR severity,^20^, ^21^, ^22^ especially patients with NYHA functional class IV, biventricular failure, or coexisting severe tricuspid regurgitation at baseline. In patients undergoing TAVI, it has already been shown that DC might be a valid parameter to optimize patient selection before intervention.^9^

Accordingly, our findings are consistent with current studies indicating that DC provides prognostic value in patients with valvular heart disease.^9^ DC of heart rate reflects autonomic nervous system function. Reduced DC mirrors compromised cardiac vagal modulation and may be correlated with disease severity, regardless of the underlying cause.^13^ In addition, Bauer et al. and Eick et al. demonstrated the association of impaired autonomic function with increased mortality.^10^, ^11^ The MitraScore was recently postulated as a simple prediction algorithm based on factors such as age, Hgb levels, glomerular filtrate rate, comorbidities, or high doses of diuretics.^23^ However, further validation studies are currently missing. DC can be easily and noninvasively determined by fully‐automated software calculations of ECG signals from either heart rhythm monitoring or 24h‐Holter ECGs. DC does not rely on drug dosages, laboratory findings, or cardiac imaging and is therefore user independent. However, it should be considered that the DC can be altered by various factors such as antiarrhythmic drugs.

Chronic severe MR affects hemodynamics and can induce severe remodeling in the left atrium (LA) and left ventricle (LV) due to volume overload and dilatation of the LA.^24^ Atrial remodeling is known to alter atrial excitability and conduction. This favors the occurrence of AF and atrial flutter. It has already been shown that electroanatomical changes in the surface ECG occur after TEER.^25^ There is also evidence that LA and LV remodeling is reversible when MR severity reduced to at least 2+ by TEER.^26^ An interaction of the sympathetic and vagus activity in the heart after TEER is therefore conceivable and could support our observation regarding the inverse correlation between the severity of the residual MR and DC. This assumption is supported by the correlation of cardiac distress markers such as atrial natriuretic peptide (ANP) and high‐sensitivity cardiac troponin‐I (hs‐cTnI) with cardiovascular death after TEER.^27^

Our study is limited due to the small sample size and the single center retrospective assessment. Furthermore, only patients with sinus rhythm were enrolled in this study. Due to the frequent presence of AF in patients with valvular heart disease, numerus patients were excluded at baseline.

## 5 CONCLUSION

In summary, DC of heart rate is associated with 1‐year mortality after TEER. Therefore, DC might be a useful additional tool to identify patients at risk and in need for close follow‐up. Correlation between DC and residual MR suggests a relation of autonomic modulation and anatomical remodeling. However, large‐scale studies are required to validate the current findings.

## AUTHOR CONTRIBUTIONS

**Lars Mizera**: Drafting of the manuscript, data collection, study conception. **Dominik Rath**: Drafting of the manuscript, study conception, assessment of data. **Jürgen Schreieck**: Data collection, critical revision. **Peter Seizer**: Data collection, statistical analysis. **Meinrad P. Gawaz**: Data collection, critical revision. **Christian Eick**: Drafting of the manuscript, data collection, statistical analysis. All authors critically revised the manuscript and approved the manuscript.

## CONFLICT OF INTEREST STATEMENT

The authors declare no conflict of interest.

## Supporting information

Supporting information.

## Data Availability

The datasets analyzed during the present study are available from the corresponding author on reasonable request.
