# Supplementary figures and images for: Deceleration capacity of heart rate predicts 1‐year mortality in patients undergoing transcatheter edge‐to‐edge mitral valve repair
Source: Clin Cardiol. 2023 Mar 22;46(5):529–34. doi: 10.1002/clc.24007 (PMC10189076; doi:10.1002/clc.24007)

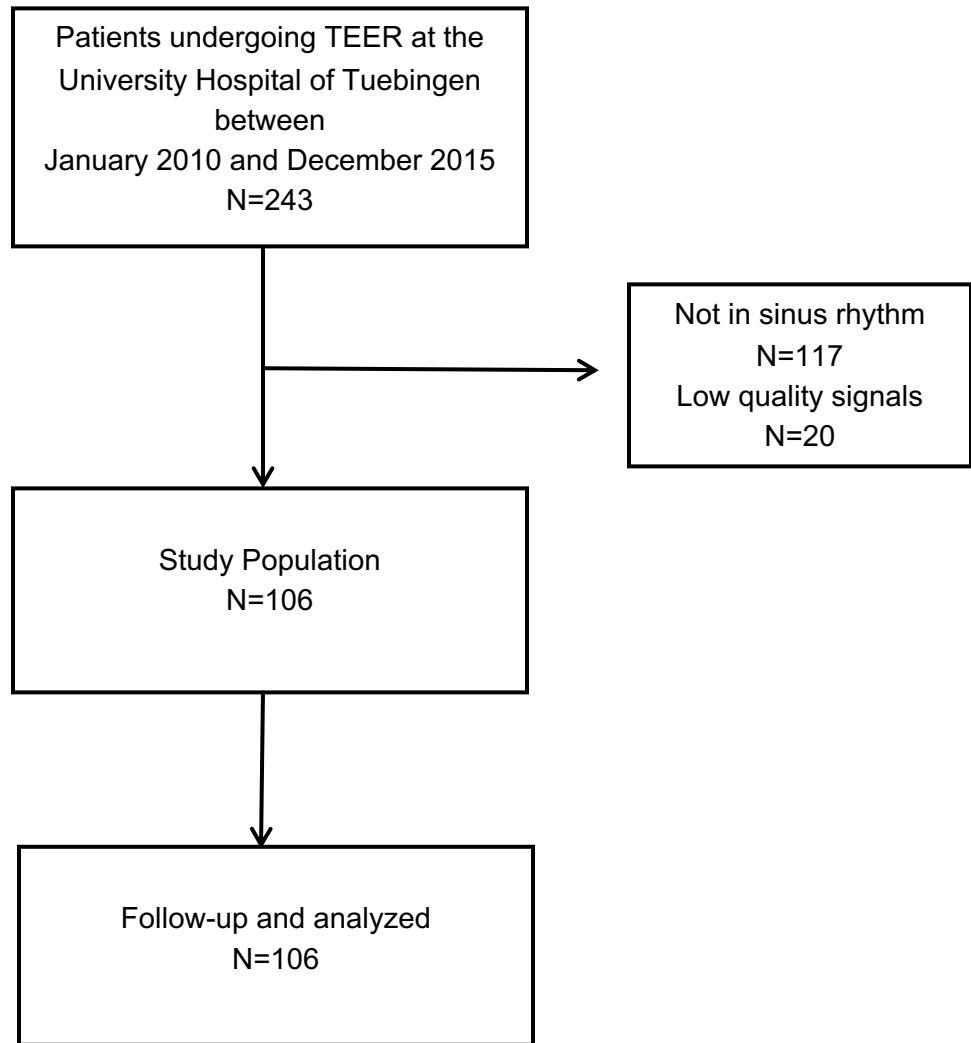

**Supplementary file** Flowchart of study inclusion

Supplement: Supplementary file 1 — Supporting information. [file CLC-46-529-s001.pdf]
